# Long-read RNA sequencing reveals full-length HERV transcripts and novel ERVE-4 isoforms with potential immunotherapeutic relevance in clear cell renal cell carcinoma

**DOI:** 10.3389/fmolb.2026.1941192

**Published:** 2026-10-29

**Authors:** Elena A. Cherkasova, Thomas Hill, Stefan Barisic, Long Chen, Jamie Diemer, Neelam Redekar, Richard W. Childs

**Affiliations:** 1 Laboratory of Transplantation Immunotherapy, Cellular and Molecular Therapeutics Branch, National Institute of Heart, Lungs, and Blood Institute, National Institutes of Health, Bethesda, MD, United States; 2 Integrated Data Sciences Section, Research Technologies Branch, National Institute of Allergy and Infectious Diseases, National Institutes of Health, Bethesda, MD, United States; 3 Department of Medicine, Duke University, Durham, NC, United States

**Keywords:** bioinformatics workflow, human endogenous retrovirus, long-read RNA sequencing, renal cell carcinoma, retrotranscriptome, splicing

## Abstract

**Introduction:**

Human endogenous retroviruses (HERVs) are increasingly recognized as sources of tumor-associated antigens and potential targets for cellular immunotherapy. However, their repetitive sequences and noncanonical splicing patterns complicate accurate transcript annotation using common short-read RNA sequencing. This study aimed to improve HERV transcriptome annotation using long-read RNA sequencing.

**Methods:**

We developed a bioinformatics workflow for comprehensive annotation of HERV expression using long-read RNA sequencing, with validation using short-read RNA sequencing. We applied this workflow to ERVE-4, a HERV element previously shown to be selectively expressed in clear cell renal cell carcinoma (ccRCC) and to encode immunogenic peptides currently under clinical investigation as targets for cellular immunotherapy.

**Results:**

Long-read sequencing captured full-length HERV transcripts, improved mapping across repetitive regions, and enabled reconstruction of HERV isoforms. The workflow identified hundreds of HERV loci in long-read datasets absent from corresponding short-read annotations. Analysis of ERVE-4 revealed previously uncharacterized isoforms, whose expression was validated across multiple ccRCC tumors using RT-dPCR.

**Discussion:**

Long-read RNA sequencing improves the resolution and completeness of HERV transcriptome annotation. Novel ERVE-4 isoforms substantially expand the known antigenic coding potential of ERVE-4 possibly implying discovery of novel peptide targets for immunotherapy in ccRCC.

## Graphical Abstract

**Figure:**
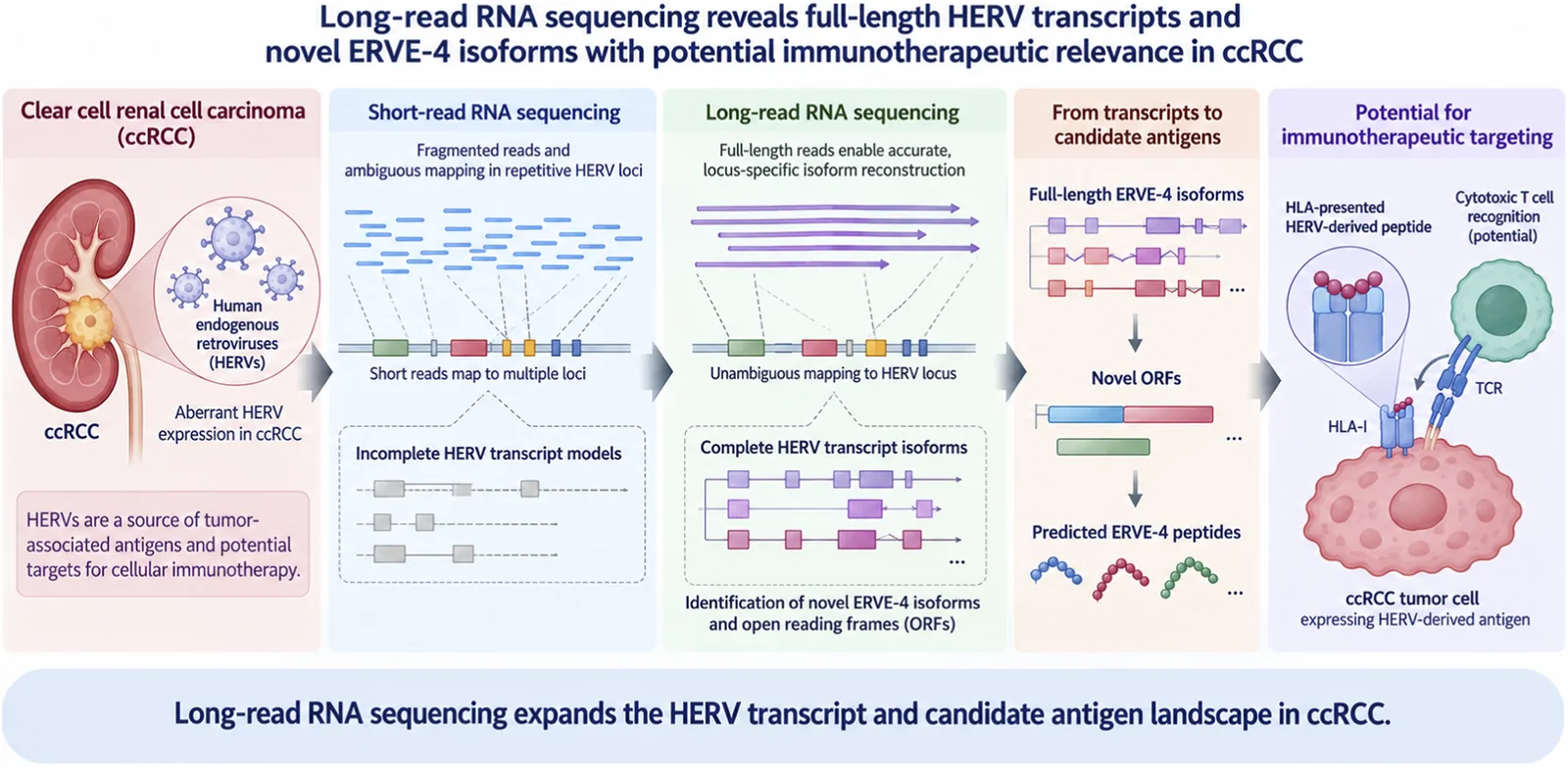
This schematic diagram outlines the central claims of the manuscript. The visual layout and individual illustrative elements of this figure were generated using ChatGPT in September, 2026 based on text prompts specifying an abstract of the manuscript. The final output was subsequently reviewed, verified, and modified by the authors to ensure factual accuracy and alignment with the experimental data. The authors retain full accountability for the integrity of this visual material.

## Introduction

Human endogenous retroviruses (HERVs) are remnants of ancient retroviral infections that became fixed in the primate germline tens of millions of years ago and now comprise approximately 8% of the human genome. Although mutations and epigenetic silencing have inactivated most HERV loci to prevent viral replication, numerous studies have demonstrated that HERVs can be aberrantly expressed in cancers, autoimmune diseases, neurodegenerative disorders, and neuropsychiatric conditions (Cherkasova et al., 2024; Duarte et al., 2024; Kury et al., 2018). In oncology, the youngest and most intact members of the HERV-K family have been studied extensively for their expression patterns and potential immunogenicity (Mazumder et al., 2025; Stricker et al., 2023; Garcia-Montojo et al., 2018).

Our group previously identified a HERV type E element (designated CT-RCC HERV-E or ERVE-4 according to HGNC nomenclature) that is selectively expressed in most clear cell renal cell carcinomas (ccRCC) and transcriptionally regulated by HIF-2α (Takahashi et al., 2008; Cherkasova et al., 2011). We demonstrated that ERVE-4 encodes immunogenic peptides capable of eliciting T cell responses (Takahashi et al., 2008; Cherkasova et al., 2016). Specifically, we isolated a CD8^+^ T cell clone from a patient with immune-mediated regression of ccRCC following allogeneic stem cell transplant, mapped its recognition of an HLA-A11–restricted ERVE-4–derived peptide (CT-RCC-1), cloned the respective full-length T cell receptor (TCR), and showed that ERVE-4–targeted TCR-engineered T cells mediated ccRCC tumor killing *in vitro* and *in vivo* (Takahashi et al., 2008; Barisic et al., 2024). These discoveries led to a first-in-human phase I clinical trial (NCT03354390), which demonstrated the feasibility and safety of adoptive transfer of ERVE-4–specific TCR-transduced T cells in patients with metastatic ccRCC (manuscript in preparation).

The sequencing of the human genome has contributed greatly to HERV studies, resulting in improved genomic mapping and classification of HERV elements. Despite these advances, the repetitive nature of HERVs and the high sequence homology within families continue to challenge comprehensive annotation of the HERVs and complicate accurate transcript mapping using short-read next-generation sequencing (NGS) platforms (Pasternack et al., 2025; Kitsou et al., 2024; Pisano et al., 2020). Although short-read-based RNA sequencing has provided valuable insights, it often cannot distinguish full-length HERV transcripts or resolve novel isoforms. Long-read sequencing technologies, by contrast, offer the ability to span entire proviral transcripts including poly(A) tails, accurately assign reads to their genomic loci, and uncover previously unrecognized spliced transcript variants. However, standardized computational tools for retrotranscriptome analysis based on long-read sequencing are not currently available.

Here, we present a bioinformatic workflow for PacBio Iso-Seq RNA sequencing, coupled with Telescope-based annotation, to systematically and accurately characterize the HERV transcriptome in ccRCC (outlined in Supplementary Figure S1). By comparing Illumina short-read and PacBio long-read RNA sequencing datasets generated from ccRCC cell lines, we found that long-read sequencing dramatically improved mapping resolution and revealed hundreds of HERV loci absent from corresponding short-read annotations. Importantly, analysis of long-read RNA sequences enabled the full reconstruction of HERV isoforms, which is impossible to achieve with short-read datasets alone. Notably, this approach uncovered previously uncategorized ERVE-4 isoforms, which we validated across numerous ccRCC tumors using Reverse Transcription digital Polymerase Chain Reaction (RT-dPCR). These newly defined isoforms broaden the ERVE-4 antigenic landscape, with potential to yield unique peptide targets for future TCR- and CAR-T-based immunotherapy in kidney cancer.

## Materials and methods

### Cell lines

We used eleven different human ccRCC cell lines (RCC1 – RCC11), established from surgically resected tumors at the NIH Clinical Center (protocols 04-H-0012, 97-C-0147, and 97-H-0196). We also used ERVE-4 knockout (KO) ccRCC cell lines, generated by depleting the entire ERVE-4 locus using CRISPR-Cas9. All tumor cells were cultured in RPMI 1640 (Gibco, Grand Island, NY, United States) supplemented with 10% heat-inactivated FBS (Sigma-Aldrich, St. Louis, MO, United States).

### Generation and validation of ERVE-4 KO ccRCC cell lines

A CRISPR-Cas9 dual sgRNA targeting strategy was used to delete the entire ERVE-4 genomic region from the genomes of four ccRCC tumor cell lines that expressed ERVE-4 (ccRCC1, ccRCC2, ccRCC3, and ccRCC4). To mitigate the risk of off-target effects on homologous HERV elements, sgRNAs were strategically designed to target flanking genomic regions upstream and downstream of the ERVE-4 long terminal repeats (LTRs) (5′-CCUAGGCAAAAUCGGCAUAG-3′ and 5′-ACUGUUCCUGCUACACCUUC-3′, respectively). The sgRNAs along with Cas9 (Synthego, Redwood City, CA, United States) were introduced into the ccRCC cell lines by electroporation using MaxCyte ATX machine (MaxCyte, Rockville, MD, United States). For isolation of edited cells, we knocked in a truncated CD19 (tCD19) into the same gene edited locus using adeno-associated virus (AAV) as donor templates. Edited ccRCC cells were enriched by CD19 magnetic microbeads (Miltenyi Biotec, Gaithersburg, MD, United States), resulting in CD19 expression of over 96%. To isolate a population of cells that had homogenous ERVE-4 knockout, we single-cell sorted and then expanded CD19^+^ ccRCC cells. Genomic DNA from numerous selected clones of each of the aforementioned cell lines was extracted using PureLink Genomic DNA Mini kit (Invitrogen, Waltham, MA, United States) and PCR analysis was performed using ERVE-4 KO-Forward (5′- TCATTCAATGCTACTATGAATAACTTCAGG -3′) and ERVE-4 KO-Reverse (5′-AAATATGTGTAAACTTGGGACTTCAGCATA–3′) primers to check for ERVE-4 knockout. PCR products were visualized by gel-electrophoresis and unveiled both monoallelic and biallelic knockout of ERVE-4. The biallelic knockout clones were further expanded. The RT-qPCR with primers specific for ERVE-4 transcripts (Takahashi et al., 2008; Cherkasova et al., 2016) and RNA sequencing were used for validation of ablation of ERVE-4 expression in the expanded ERVE-4 KO clones (manuscript in preparation). The absence of the ERVE-4-derived CT-RCC-1 peptide in the ERVE-4 KO clone was demonstrated via targeted parallel reaction monitoring mass spectrometry (PRM-MS) as described in Barisic et al., 2024 (10).

### RNA processing and sequencing

We isolated total RNA from cultured cells using a PureLink RNA Mini Kit (Invitrogen, Waltham, MA, United States) according to the manufacturer’s protocol. Following QC of the RNA, we prepared poly(A)-selected mRNA libraries and sequenced them on the Illumina MiSeq platform (Saffron Walden, UK), yielding ∼20 million paired-end reads per sample. We generated libraries with the SMRTbell Prep Kit v3.0 (PacBio, Menlo Park, CA, United States) and performed long-read RNA sequencing using the PacBio Sequel II platform with the Iso-Seq workflow. Across samples, the average yield was 3.48–3.68 million circular consensus sequencing (CCS) reads with a mean quality score > QV38.2. Both the PacBio IsoSeq and Illumina MiSeq reads are available on GEO (accessions: GSE318402).

### RNA sequencing data processing and transcript annotation

We processed PacBio long-read RNA sequencing data using the IsoSeq3 pipeline (https://github.com/ylipacbio/IsoSeq3). Specifically, we pre-processed PacBio circular consensus sequencing (CCS) reads to remove cDNA primers and barcodes, then refined the reads to remove poly(A) tails and concatemer sequences to produce full-length non-concatemer (FLNC) sequences. We merged and clustered the FLNC sequences from all samples to define consensus transcript isoforms and clustered sequences from individual samples separately to obtain non-redundant isoforms. We then aligned each set of transcripts to the GRCh38 (hg38) human reference genome and classified all sets of transcript isoforms using the PacBio transcript toolkit Pigeon (Supplementary Figure S1) (https://github.com/marianoguera/SMRT-Analysis). This classification utilized a modified hg38 reference human genome GENCODE annotation, which contained the HERV reference annotation generated by Telescope (Bendall et al., 2019). We combined the classified isoforms across the merged set and three individual libraries using gffcompare (https://github.com/gpertea/gffcompare) to generate complete set of isoforms detected in three ccRCC samples (Supplementary Figure S2).

From this complete set of annotated transcripts (including both GENCODE genes and HERVs), we extracted the consensus transcript sequences for each gene and HERV isoform using gffread (https://github.com/gpertea/gffread), and aligned the FLNC sequences from each sample to the extracted consensus transcripts using pbmm2, with the ‘isoseq’ preset parameters. Next, we estimated the number of reads mapping to each transcript using SAMtools and calculated the transcripts per kilobase per million (TPM) per transcript in each sample using R. To ensure we did not miss any reference Telescope HERV loci, we extracted the reads that did not align to any isoforms and remapped them to the Telescope HERV transcript sequences following the previously described method. We then removed all isoforms annotated as genes and retained only HERVs previously mapped by Telescope (“known”) and newly found HERVs (“novel”) to represent total HERV isoforms identified in our ccRCC libraries. These known and novel HERV isoforms are summarized in this study to represent HERV landscape in ccRCC cells (Figure 1). Using the total generated annotation (both GENCODE genes and HERVs), we processed Illumina short-read data for each sample with the RNA-seek pipeline (https://github.com/skchronicles/RNA-seek) to quantify the expression of each HERV. Briefly, this pipeline includes quality control by FastQC v0.11.9, Preseq v2.0.3, Picard v2.17.11, FastQ Screen v0.9.3 (Wingett and Andrews, 2018), Kraken2 v2.0.8 (Wood et al., 2019), QualiMap (Garcia-Alcalde et al., 2012), and RSeQC v2.6.4 (Wang et al., 2012). The pipeline trims adapters and low-quality reads with Cutadapt v1.18 (Martin, 2011). It then aligns trimmed reads to the GENCODE GRCh38. p13 reference genome using the splicing-aware aligner STAR v2.7.6a (Dobin et al., 2013) in two-pass mode, against both the genome and the extracted transcriptome. Finally, it quantifies the expression of Illumina short-read RNA sequencing datasets against the transcriptome using RSEM v1.3.3 (Li and Dewey, 2011).

**FIGURE 1:**
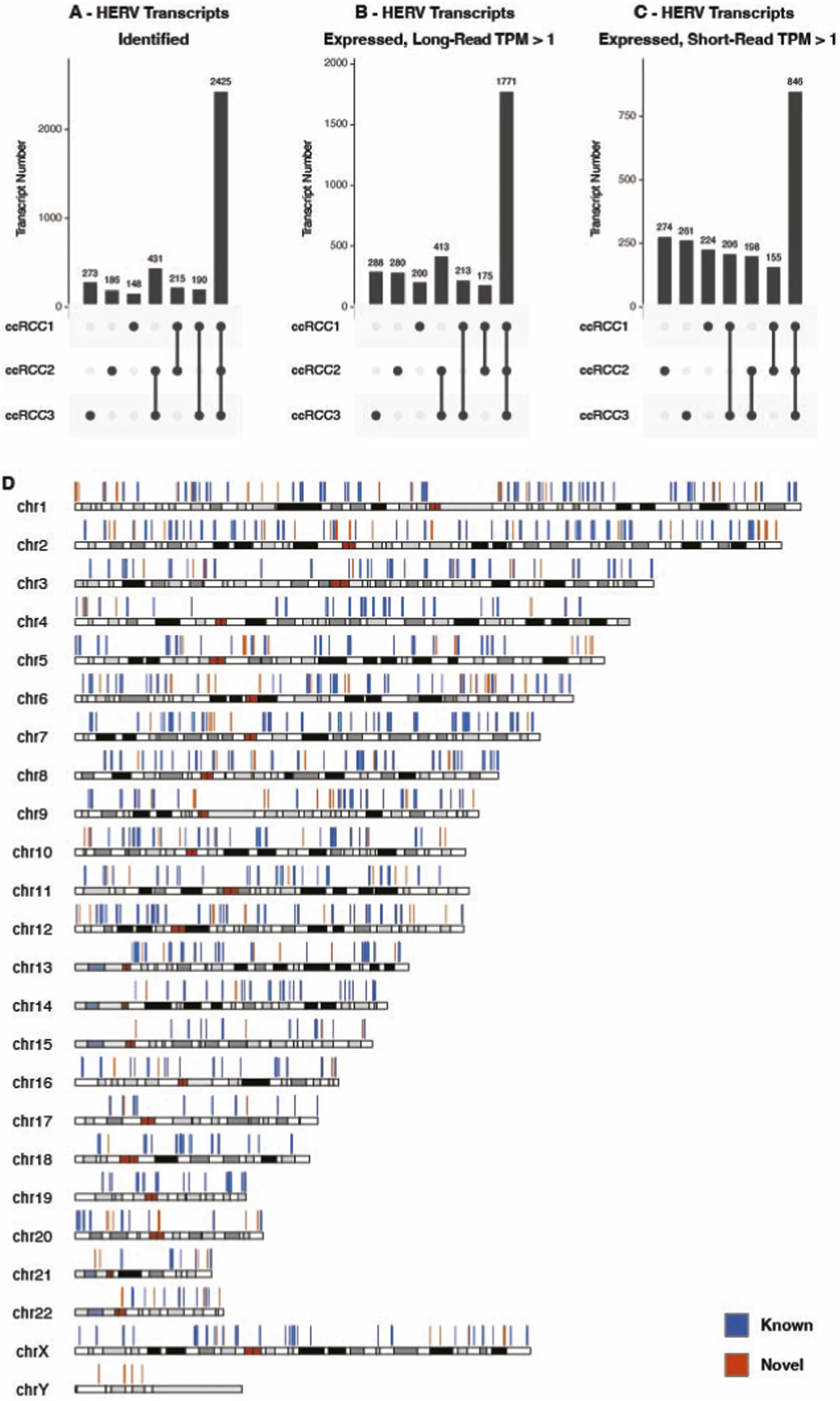
HERV loci found to be expressed in three ccRCC cell lines. **(A)** number of full-length HERV transcripts identified; **(B)** number of full-length HERV transcripts found to be expressed (TPM > 1) in long-read datasets. **(C)** number of HERV transcripts found to be expressed (TPM > 1) in short-read datasets. **(D)** Genomic landscape of HERVs in ccRCC cells identified by analysis of long-read and short-read RNA sequencing datasets of three ccRCC cell lines. Known HERV loci are marked in blue and novel in red.

We then filtered the annotations to remove the GENCODE genes, and to remove HERV transcripts which showed no expression in any RNA sequencing dataset and then summed the transcript expression to find the total expression per separate HERV in each ccRCC cell line. We also considered each HERV based on their presence and absence in each sample separately, and if their expression in TPM was greater than 1.0 in either the long-read or short-read RNA sequencing reads for each sample. We generated genome-wide maps of HERV expression for three ccRCC cell lines analyzed by short-read RNA sequencing platform using karyoploteR (Gel and Serra, 2017). All statistical analyses were conducted in R.

### Targeted sequencing of splice junction regions

To resolve ambiguous regions surrounding second splice site junction in transcript ERVE-4 –pol-2X-env, we designed isoform-specific primers for PCR (forward 5′-CAACAGTACCTAACCCGTACAC-3’; reverse 5′-GGCCTTCTGGACTGGAATTAT-3′) and examined the resultant gel-purified PCR products from three ccRCC samples (ccRCC1, ccRCC2, and ccRCC3) by targeted sequencing. We prepared libraries using the Nextera XT kit (Illumina) and sequenced them on a MiSeq Nano platform. Following the sequencing of the amplicons, we used DADA2 v1.16 within R v4.4.2 to merge the paired-end sequences and remove chimeras. We aligned merged reads to the ERVE-4 genomic sequence using minimap2 v2.28 and generated consensus sequences with SAMtools v1.21. Finally, we performed multiple sequence alignment with MAFFT v7.526 and generated sequence logos using ggseqlogo to plot the consensus sequence of the splice site (hg38 genomic coordinates: Chr.6q 88 668 524/88 669 078).

### Reverse Transcription-digital PCR (RT-dPCR)

We used the QIAcuity OneStep Advanced Probe kit (Qiagen, Hilden, Germany) to quantify RNA targets in one combined RT-dPCR reaction per the manufacturer’s protocol. Using a sample input of 50 ng of RNA, we performed reactions using QIAcuity One instrument (Qiagen) and QIAcuity nanoplates with 8.5k partitions. We applied the following cycling parameters: RT reaction at 50 °C for 40 min, heat activation at 95 °C for 2 min, followed by 40 cycles of denaturation (95 °C, 15s) and combined annealing/extension (60 °C, 30s) with default QIAcuity imaging parameters for each dye channel. We then analyzed the resulting data analysis using QIAcuity Software Suite (Qiagen).

We designed custom primers/probes assays specific for the junction regions between the splice donor (SD) site SD1 and different splice acceptor (SA) sites using the NCBI Primer-BLAST and synthesized by Integrated DNA Technologies (IDT, Coralville, IA, United States). The sequences of primers and probes used for validation and quantification of different ERVE-4 transcript variants in RT-dPCR are listed in Table 1. We purchased pre-designed validated reference gene assays for HPRT and PPIH house-keeping genes from IDT.

**TABLE 1:** Primers and probes sequences used in RT-dPCR specific to different splicing regions found in four ERVE-4 transcript variants.

ERVE-4 transcript	SD/SA	Primers (forward and reverse) and probes (5′-3′)
ERVE-4-pol-2X-env	SD1/SA1	F: CGATTGGAGTCTGGACATCTG R: CACCGGGTTGGCATTCA P: TGCGGCTTGCTGCATCATGTTC
CT-RCC-env	SD1/SA2	F: ATCGGCGATTGGAGTCTGG R: TCCACCGTGGCCACAAA P: TGGAACATGGTGATCGAGTGTGGA
ERVE-4-pol-env-A	SD1/SA3	F: CATGTACATCGACAACCTCCT R: CTTGGACACCTTATACCCATAGTC P: TCAAAGGAACAGACGCCCTGCTT
ERVE-4-pol-env-B	SD1/SA4	F: TTGAACACCGGGAAGGAATC R: GGGCTATGTTCCAGTCTTTGA P: CCAGATGTCCAGACTCCAATCGCC

## Results

### HERV transcriptome is consistent across three ccRCC cell lines

To study the totality of all HERV transcripts in ccRCC cell lines, we used long-read (PacBio) RNA sequencing data to assemble and classify isoforms from three independent ccRCC cell lines. Then we quantified expression of HERV genes and isoforms using short-read (Illumina) RNA sequencing data (Pipeline shown in Supplementary Figure S1). We assembled full-length transcripts from individual ccRCC lines and from a dataset merged across all three samples to ensure the capture of the rarest transcripts. Overall, we identified 182 504 full-length transcripts in the combined dataset with more than 90K transcripts assembled in each separate ccRCC library (Table 2, ccRCC1 = 90 837, ccRCC2 = 90 769, ccRCC3 = 95 941). Structural classification suggested that the transcripts assembled with combined data captured higher number of non-canonical transcripts, including novel isoforms, compared to transcripts assembled in three individual libraries (Supplementary Figure S2). Because of this, we excluded GENCODE genes and focused our analysis on a non-redundant consolidated ccRCC transcriptome set of 4 197 HERV transcript isoforms, of which 99.07% of the HERV transcripts (n = 4 158) showed complete, exact match of exon-intron junction pattern across three different ccRCC lines (Supplementary Figure S3, mismatches shown in Supplementary Table S1).

**TABLE 2:** IsoSeq assembly and classification summary.

Total full-length transcripts assembled		ccRCC1 (%)	ccRCC2 (%)	ccRCC3 (%)	Combined (%)
		90,837	90,769	95,941	182,504
IsoSeq	Structural characteristics				
	Longest isoform	14,296	14,901	14,288	14,901
	Median isoform length	2887	2722	2887	2848
	Total genes	14,796	17,015	17,570	23,294
	Number of isoforms/gene (median)	4	3	3	3
	Exons/isoform (median)	9	8	8	8
	Exons/isoform (max)	106	89	86	106
	Coding isoforms	87,325	86,738	92,135	174,052
	Non-coding isoforms	3512	4031	3806	8452
Pigeon classification (count, %)	Structural categories				
	Antisense	116 (0.1)	148 (0.2)	134 (0.1)	358 (0.2)
	Full-splice_match	36,865 (40.6)	36,704 (40.4)	38,313 (39.9)	60,373 (33.1)
	Fusion	456 (0.5)	536 (0.6)	664 (0.7)	1380 (0.8)
	Genic	47 (0.1)	77 (0.1)	91 (0.1)	179 (0.1)
	Incomplete-splice_match	16,460 (18.1)	16,717 (18.4)	17,177 (17.9)	37,775 (20.7)
	Intergenic	184 (0.2)	199 (0.2)	167 (0.2)	476 (0.3)
	moreJunctions	7 (0.02)	21 (0.01)	19 (0.02)	38 (0.02)
	novel_in_catalog	22,622 (24.9)	22,722 (25.0)	24,659 (25.7)	47,958 (26.3)
	novel_not_in_catalog	14,080 (15.5)	13,645 (15.0)	14,717 (15.3)	33,967 (18.6)
HERV isoforms	HERV isoforms				
	Total HERV genes	960	969	969	1334
	Total HERV isoforms	2978	3257	3233	5034
	Number of isoforms/gene (median)	1	1	2	1
	Exons/isoform (median)	10	10	9	9

We next sought to quantify the expression of all HERV transcripts in both long and short-read data. Long-read isoforms exhibited a higher percentage of uniquely mapped reads (95%–97%) than short reads mapping (83%–85%; Table 2). This pipeline identified 2 978–3 257 HERV transcripts across samples, belonging to 961–986 HERV loci (Figure 1A; Supplementary Figure S4), of which 678–689 were previously described by (Bendall et al., 2019) in normal tissues and various tumors excluding ccRCC. The majority (2 359–2 685) of these transcripts (originating from 643 to 663 known HERV loci and 250–271 novel HERV loci) were expressed at high levels (Figure 1B, TPM > 1; Supplementary Figure S4).

Comparatively, the short-read RNA sequencing identified 1 428–1 497 HERV transcripts in three ccRCC cell lines, from 618 to 635 HERV loci, expressed at high levels (Figure 1C, TPM > 1; Supplementary Figure S4), of which 528–536 were previously described (Bendall et al., 2019). Overall, we found 1 400–1 613 HERV transcripts and 286–326 HERV loci in the long-read RNA sequencing data that were entirely undetected by the short-read sequencing platform (Supplementary Figure S5). Notably, we found all HERV transcripts detected in short-read data within the long-read datasets (Supplementary Figure S6). This underscores the superior ability of long-read RNA sequencing to comprehensively characterize the retrotranscriptome.

In total, long-read RNA sequencing identified 1 034 distinct HERVs expressed in at least one of the three ccRCC cell lines examined. Similar to previous findings (Bendall et al., 2019), certain genomic regions and chromosomes exhibit low or high HERV density potentially resulting from insertional biases during human evolution (Figure 1D).

### Discovery of novel ERVE-4 transcript variants

We next focused on ERVE-4, a HERV element previously shown to be selectively expressed in ccRCC and to encode immunogenic antigens with clinical relevance in kidney cancer (Takahashi et al., 2008; Cherkasova et al., 2011; Cherkasova et al., 2016; Barisic et al., 2024; Ficial et al., 2021). Coverage analysis revealed comparable overall expression patterns across the ERVE-4 locus in long-read sequencing of all three samples, as well as similar patterns in short-read sequencing data (Supplementary Figure S7).

Focusing on long-read isoforms prevailing in 3 cell lines examined, we found four full-length ERVE-4 transcripts; three singly and one doubly spliced variants (Figures 2, 3). Sequence comparison revealed that all novel transcripts have the same splice donor site (SD1) in the 5′LTR region as the previously identified CT-RCC-8, CT-RCC-9 and CT-RCC-Env transcripts (Takahashi et al., 2008; Cherkasova et al., 2016) (genomic coordinates of SD and SA sites on chr.6q are shown in Figure 3). One of four isoforms represents previously known transcript CT-RCC-Env (Cherkasova et al., 2016) and the remaining three are novel ERVE-4 transcript variants (Figure 3). Three singly spliced variants, including previously known CT-RCC-Env, encode for different portions of *pol* gene and the whole *env* gene. Specifically, the novel ERVE-4-pol-env-A variant covers a major part of the *pol* gene in addition to the *env* gene sequence. The newly identified transcript ERVE-4-pol-env-B is only 18 nucleotides longer in 3′-part of the *pol* gene than CT-RCC-Env transcript. The longest and doubly spliced transcript variant encodes for a small 3′-region of the *gag* gene, the entire *pro* and *pol* genes, and a partially truncated *env* gene, resulting from the second splicing event (Figure 3). All four transcripts encode open reading frames (ORFs) in the same reading frame. The novel ERVE-4-pol-env-A and the doubly spliced transcript ERVE-4-pol-2X-env have additional coding capacity in *gag*, *pro* and *pol* genes in comparison to previously known ERVE-4 transcripts. Our discovery of novel transcripts with extended coding capacity could lead to the identification of new ERVE-4-derived antigens for future immunotherapy strategies in the treatment of ccRCC.

**FIGURE 2:**
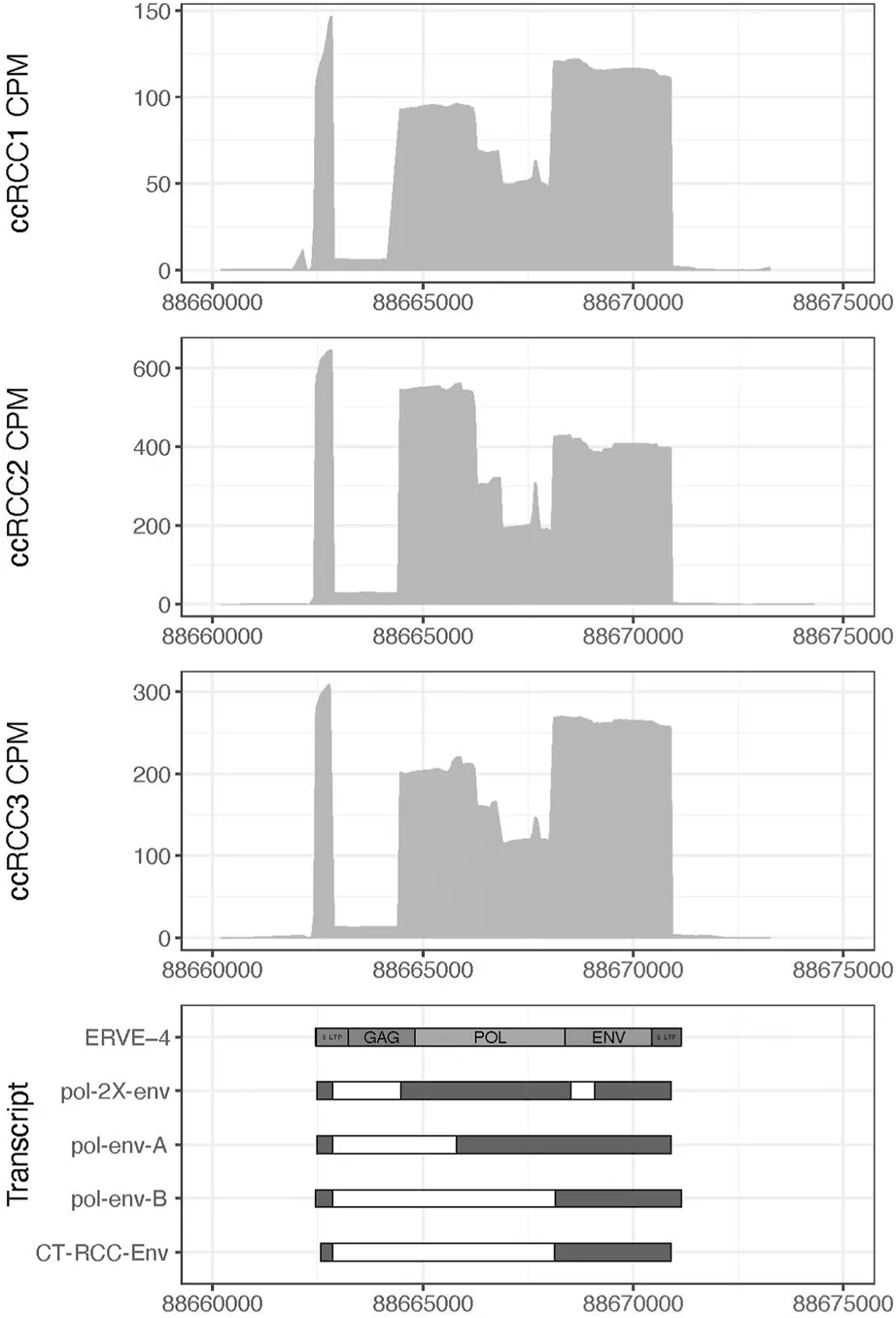
Transcriptional landscape of ERVE-4 element defined by long-read sequencing of three ccRCC cell lines. **(A)** Comparison of the coverage of ERVE-4 genomic region in CPM in three ccRCC samples. Axis x, genomic coordinates; axis y, CPM value per base. **(B)** Schematic representation of four ERVE-4 isoforms found to be expressed across all three samples. Black bars represent exons and white bars introns.

**FIGURE 3:**
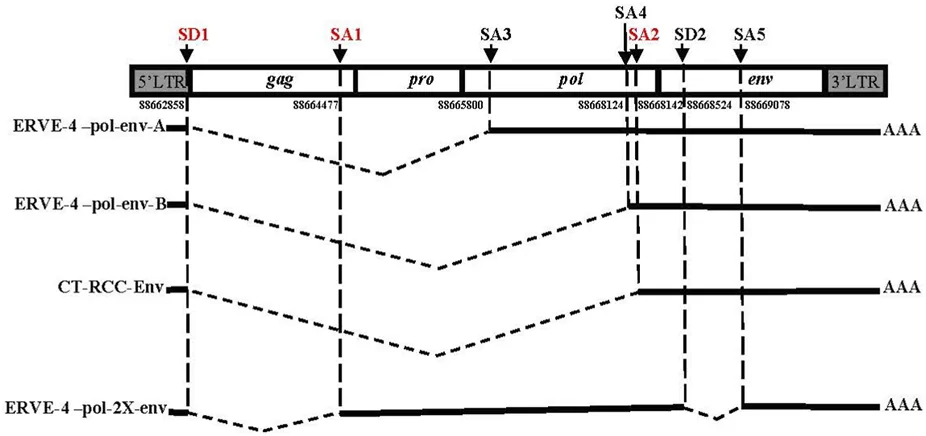
Schematic representation of ERVE-4 proviral structure and its singly spliced and doubly spliced transcripts. Genomic coordinates of SD and SA sites are shown. Previously known splicing sites SD1, SA1, and SA2 are labeled according to our previous publication (Cherkasova et al., 2016) and marked in red.

### Validation of novel ERVE-4 transcripts expression across ccRCC cell lines by RT-dPCR

To analyze the presence of the newly identified ERVE-4 isoforms in different ccRCC tumors, we performed RT-dPCR using four custom primers–probe assays (Table 1) designed to specifically detect both, previously described (SD1/SA1 and SD1/SA2) and newly identified (SD1/SA3 and SD1/SA4) splice junctions in four ERVE-4 isoforms. As specificity controls, we included ERVE-4-negative ccRCC cell lines in which CRISPR/Cas9 was used to delete the entire ERVE-4 locus (named ccRCC1-ERVE-4 KO, ccRCC2-ERVE-4 KO, and ccRCC3-ERVE-4 KO).

RT-dPCR of total RNA from all three parental wild-type ccRCC lines yielded robust isoform-specific signals, whereas we detected no amplification in any ccRCC ERVE-4 KO variants, confirming the high specificity of all assays (Table 3). We next extended validation to nine additional ccRCC cell lines (ccRCC4–ccRCC11), including an ERVE-4 knockout derivative of ccRCC4 (ccRCC4-ERVE-4 KO). Despite variable ERVE-4 expression levels between cell lines, we consistently detected all four isoforms in all nine wild-type lines but not in the ccRCC4 ERVE-4 KO, providing strong evidence that these isoforms are universally expressed across different ccRCC tumors (Table 3).

**TABLE 3:** RT-dPCR using primers/probe PCR assays targeting SD1 and different SA sites in four ERVE-4 isoforms. ERVE-4 expression was normalized to geometric mean of expression of HPRT and PPIH genes. All experiments were performed in triplicates.

Cell line	SD1_SA1	SD1_SA2	SD1_SA3	SD1_SA4
ccRCC1	1.74	5.97	1.93	6.32
ccRCC1 ERVE-4 KO	0	0	0	0
ccRCC2	0.5	0.67	0.64	0.68
ccRCC2 ERVE-4 KO	0	0	0	0
ccRCC3	0.71	0.93	0.93	0.92
ccRCC3 ERVE-4 KO	0	0	0	0
ccRCC4	0.43	1.43	0.43	1.07
ccRCC4 ERVE-4 KO	0	0	0	0
ccRCC5	0.91	2.99	1.21	3.12
ccRCC6	0.65	0.37	0.36	0.36
ccRCC7	0.27	0.31	0.26	0.29
ccRCC8	0.02	0.09	0.02	0.09
ccRCC9	0.01	0.04	0.02	0.05
ccRCC10	0.58	0.72	0.67	0.75
ccRCC11	0.1	0.14	0.1	0.14

Interestingly, relative isoform abundance varied between the tumor-derived cell lines. In five of 11 cell lines (ccRCC2, ccRCC3, ccRCC7, ccRCC10, and ccRCC11), all four isoforms were expressed at similar proportions, whereas the remaining six lines exhibited distinct expression patterns, with certain isoforms predominating (Figure 4). These data indicate that different ccRCC tumors not only express multiple ERVE-4 splice variants but also differ in their relative isoform usage, reflecting heterogeneity in ERVE-4 splicing regulation.

**FIGURE 4:**
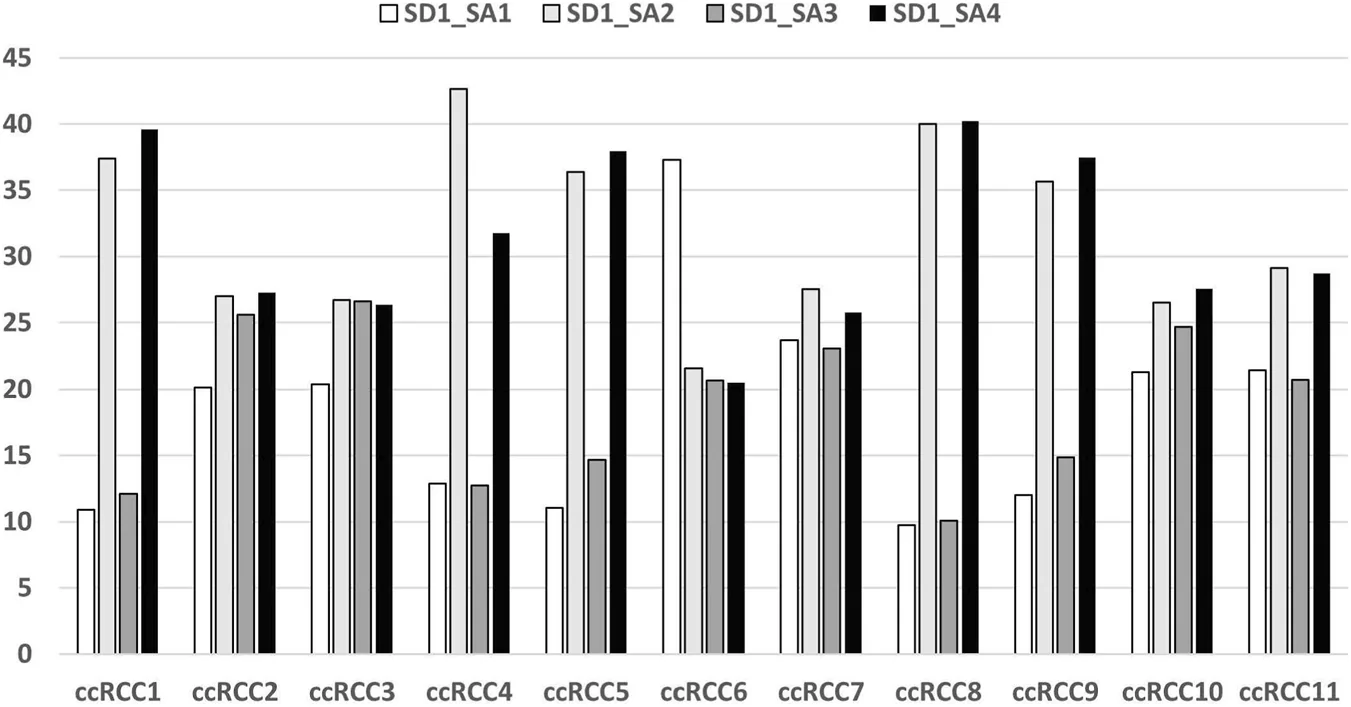
Proportions of different ERVE-4 splice variants transcripts found by RT-dPCR analysis of different ccRCC cell lines. ERVE-4 expression is normalized to geometric mean of expression of HPRT and PPIH house-keeping genes. All experiments were performed in triplicates.

## Discussion

To the best of our knowledge, this is the first study applying long-read RNA sequencing to the analysis of HERV transcriptome in cancer cells, highlighting the broader advantages of long-read technologies for investigating endogenous retroviral transcriptional landscapes. Prior reports have shown that long-read libraries provide more accurate transcript assemblies than short-read data even at high sequencing depth (Pasternack et al., 2025; Pardo-Palacios et al., 2024). Short-read methods often discard multimapping reads or misassign repetitive fragments, leading to underestimation of expression of numerous HERVs. Several bioinformatic tools have attempted to address these challenges, including RepeatMasker (Bailly-Bechet et al., 2014), RetroTector (Sperber et al., 2007), ERVmap (Tokuyama et al., 2018), RetroSnake (Kabiljo et al., 2022), WebHERV (Kruse et al., 2018), and Telescope (Bendall et al., 2019). Yet, as others have noted, the number and coordinates of annotated HERV loci vary substantially across databases, raising concerns about reproducibility. We applied Telescope in our bioinformatic workflow, which is a widely used HERV annotation tool based on a Bayesian framework for probabilistically reassigning ambiguously mapped reads (Bendall et al., 2019). In contrast to (Bendall et al., 2019), who used Telescope to short-read RNA sequencing data from the ENCODE project to profile HERV expression across thirteen major human cell types, including five cancer-derived cell lines, we here extend its application to long-read RNA sequencing data. Although kidney cancer was not included in the original Telescope studies, we found this combined approach to be powerful tool for HERV annotation of ccRCC cells. We expanded genome-wide cell type specific HERV maps previously described by (Bendall et al., 2019) by detecting numerous additional HERV elements with expression specific to ccRCC kidney cancer (Figure 1D). The increased number of reactivated HERV elements detected in this analysis of kidney cancer, exceeding those previously reported across diverse cell types and malignancies (Bendall et al., 2019), likely reflects two factors: the enhanced detection capacity of long-read sequencing, and the possibility that ccRCC, as a highly immunogenic tumor type, expresses a broader repertoire of HERV elements than other cancers. Notably, while most transcripts were consistently observed across all ccRCC cell lines, we detected minor differences in the number of annotated HERV loci (Supplementary Figure S4). These fluctuations may reflect biological heterogeneity but could also arise from alignment challenges in highly repetitive regions.

Long-read sequencing may introduce artifacts such a failure to capture truncated transcript, or ambiguous alignment within repetitive HERV loci, particularly for low-abundance isoforms supported by few reads. We therefore interpret rare isoforms cautiously, with greater confidence assigned to transcripts reproducibly detected across different samples and supported by independent short-read RNA sequencing. Overall, our findings underscore the value of long-read RNA sequencing in more precisely defining the HERV transcriptome, overcoming key limitations of short-read sequencing technologies in a) resolving full length transcripts, b) distinguishing highly similar loci, and c) accurately characterizing transcript structure. Moreover, our results demonstrate that integrating long-read sequencing with tools such as Telescope can improve locus-level annotation, enabling the detection of a greater number of HERV transcripts than is achievable with short-read RNA sequencing (Supplementary Figures S4–S6). While short-read RNA sequencing captured broad expression patterns, our long-read RNA sequencing analysis allowed for the full reconstruction of the ERVE-4 isoforms, leading to the identification of three novel variants in addition to previously described transcripts (Figures 2, 3). A limitation of this study is that long-read sequencing was performed in only three ccRCC cell lines, which may not capture the full transcriptional heterogeneity of primary tumors. Consequently, additional HERV loci and isoforms may remain unidentified. Expanded long-read profiling of additional tumor lines as well as primary ccRCC specimens will be important to define the broader repertoire of ERVE-4 transcripts.

Prior work has demonstrated that even short ORFs preserved within HERVs can produce immunogenic peptides (Schiavetti et al., 2002). More recent studies further support the role of HERVs as a source of tumor-associated antigens in cancer progression and as promising targets for cellular immunotherapy (Cherkasova et al., 2024; Wu et al., 2026; Jiang et al., 2025; Bonaventura et al., 2022; Muller et al., 2022). In this context, our identification of new ERVE-4 transcripts provides a more complete view of the antigenic repertoire of this locus. Given their restricted expression in ccRCC, these transcripts may represent compelling candidate targets for immune-based therapies in kidney cancer. We also observed variability in the relative abundance of isoforms derived from the same HERV locus across ccRCC cell lines. Although all four ERVE-4 isoforms identified in this analysis were detected in every ccRCC cell line tested by RT-dPCR, the ratios between individual isoforms varied markedly from one cell line to another (Figure 4). This variability may reflect tumor-specific regulation of splicing or transcript stability, altering tumor antigenic profiles and, consequently, tumor immunogenicity. From an immunotherapy standpoint, such heterogeneity presents both challenges and opportunities. Variability in isoform abundance across tumors may require distinct targeting strategies depending on the dominant tumor-specific isoform profile. At the same time, this more accurate identification of the endogenous retroviral transcriptome provides an opportunity to prioritize targeting of tumor antigens derived from the dominant isoform. This may ultimately improve the likelihood of a benefit from antigen-directed immunotherapy.

The therapeutic relevance of these findings is underscored by prior demonstration that ERVE-4 is selectively expressed in ∼90% of ccRCCs, absent from normal tissues, and encodes antigens recognizable by T cells (Takahashi et al., 2008; Cherkasova et al., 2011; Cherkasova et al., 2016; Barisic et al., 2024; Rooney et al., 2015). Building on this, we cloned a TCR targeting the HLA-A11–restricted ERVE-4-derived peptide and showed that TCR engineered T cells mediate tumor-specific tumor killing of ccRCC tumors *in vitro* and *in vivo*, leading to the first-in-human clinical trial of HERV-directed TCR gene-therapy for metastatic kidney cancer (Barisic et al., 2024). However, the limited prevalence of HLA-A11 in many populations highlights the need to identify additional peptides presented by more common HLA alleles. Future studies will be needed to determine whether the novel ERVE-4 ORFs in transcripts identified here are translated and contribute to the tumor antigen repertoire. Such studies could include immunopeptidomics and targeted mass spectrometry-based detection of predicted peptides to validate protein expression and presentation of peptides by different HLA molecules in ccRCC. These approaches will help define ORFs that lead to *bona fide* antigenic products.

The importance of ERVE-4 as a source of immunogenic peptides in ccRCC is further highlighted by the recent work of Lu et al. (2021), who developed a novel deep learning-based model of pMHC-TCR binding prediction network (pMTnet) to characterize the TCR-antigen binding specificities for both neoantigens and tumor-associated antigens, including self-antigens such as HERV-derived peptides. Analyzing extensive genomic data across several highly immunogenic tumor types, including ccRCC, they found that ERVE-4 exhibited the highest tumor-to-normal expression ratio among all HERVs, markedly exceeding that of other HERV families. Moreover, although neoantigens were generally more immunogenic than self-antigens, analysis of tumor antigens predicted to bind to at least one TCR showed that ERVE-4–derived antigens surpassed both neoantigens and other self-antigens in immunogenicity in ccRCC (Lu et al., 2021).

In conclusion, this study establishes long-read RNA sequencing as a superior analytic approach for HERV transcriptome analysis, as the ability to span full-length transcripts overcomes limitations of short-read sequencing, whose mapping resolution is compromised by the repetitive nature of HERV sequences. Specifically, long-read data enabled the discovery of ERVE-4 transcript variants beyond those previously identified. Future efforts to identify novel ERVE-4–derived antigens, together with TCRs and antibodies specific to them, will be critical for translating these transcriptomic insights into clinically actionable TCR- and CAR-based therapies for kidney cancer.

## Data Availability

The datasets presented in this study can be found in online repositories. The names of the repository/repositories and accession number(s) can be found below: https://www.ncbi.nlm.nih.gov/ GSE318402.

## Supplementary material

SUPPLEMENTARY FIGURE 1 Pipeline developed for HERV annotation and expression assessment in PacBio long-read and Illumina short-read RNA sequencing datasets.

SUPPLEMENTARY FIGURE 2 Summary of the ccRCC transcriptome assemblies (and combined assembly), split into counts of each classification category defined by Pigeon.

SUPPLEMENTARY FIGURE 3 Barplot quantifying the overlap of HERV transcripts across four annotations using gffCompare.

SUPPLEMENTARY FIGURE 4 Differences in the total number of HERV loci found to be expressed in three ccRCC cell lines **(A-C)**. Differences in the total number of HERV transcripts found to be expressed in three ccRCC cell lines **(D-F)**.

SUPPLEMENTARY FIGURE 5 Quantification of HERV expression in TPM using short-read (y, axis) and long-read RNA sequencing data (x, axis) and comparison of data found in three ccRCC samples. Red dots, novel HERV loci found by analysis both datasets in each sample; black dots, known HERV loci.

SUPPLEMENTARY FIGURE 6 Transcriptional landscape of HERVs in ccRCC cells identified by analysis of PacBio long-read and Illumina short-read RNA sequencing datasets of three ccRCC cell lines. The outer track is a bar chart showing the number of HERV loci in 10 Mbp windows, ranging from 0 to 100. The six inner rings show the expression levels (0 to 10 log2 FPKM of HERV loci that were expressed in three ccRCC cell lines. Moving from the outer ring to the inner ring, the samples are as follows: ccRCC1 long-read, ccRCC1 short-read, ccRCC2 long-read, ccRCC2 short-read, ccRCC3 long-read, ccRCC3 short-read RNA sequencing datasets.

SUPPLEMENTARY FIGURE 7 Comparison of the coverage of ERVE-4 genomic region in CPM from Illumina data (top panel) and PacBio (bottom panel) in three ccRCC samples. Axis x, genomic coordinates; axis y, CPM values.
