# Supplementary material for: Long-read RNA sequencing reveals full-length HERV transcripts and novel ERVE-4 isoforms with potential immunotherapeutic relevance in clear cell renal cell carcinoma
Source: Front Mol Biosci. 2026 Oct 29;13:1941192. doi: 10.3389/fmolb.2026.1941192 (PMC13754704; doi:10.3389/fmolb.2026.1941192)
Supplement: Supplementary file 1 [file Table1.docx]

| **Transcript classification** | **HERV isoform ID** | **Transcript Isoforms ID** | **Isoform detection in ccRCC libraries** |
| --- | --- | --- | --- |
| **Fully contained (c = 1)** | novelGene_MER41_15q21.2a_AS | TCONS_00040798 | ccRCC2 |
| **Potentially novel isoforms (j = 27, but 25 unique)** | novelGene_9 | TCONS_00012636 | ccRCC1, ccRCC2, ccRCC3 |
|  | novelGene_ERVL_1p34.2_AS | TCONS_00012654 | ccRCC2 |
|  | novelGene_HERVP71A_10q11.22_AS | TCONS_00018836 | ccRCC2 |
|  | novelGene_HML3_13q12.11b_AS | TCONS_00034263 | ccRCC3 |
|  | novelGene_HERVIP10FH_13q12.11_AS | TCONS_00034264 | ccRCC3 |
|  | novelGene_HERVL_14q24.2c_AS* | TCONS_00037472 | ccRCC1, ccRCC3 |
|  | HERVL74_16q24.3 | TCONS_00045787 | ccRCC2 |
|  | HERVE_19q12 | TCONS_00059550 | ccRCC1 |
|  | novelGene_HML6_19q13.41a_AS | TCONS_00059596 | ccRCC2 |
|  | HARLEQUIN_19q13.41 | TCONS_00059625 | ccRCC3 |
|  | novelGene_HERVH_2p16.1c_AS | TCONS_00068894 | ccRCC1 |
|  | novelGene_HERVW_2q31.2_AS | TCONS_00068916 | ccRCC1 |
|  | novelGene_ERVLE_2q32.1c_AS | TCONS_00068918 | ccRCC1 |
|  | novelGene_ERVLB4_2p16.3_AS | TCONS_00069017 | ccRCC2 |
|  | novelGene_4990 | TCONS_00069022 | ccRCC2 |
|  | novelGene_4981 | TCONS_00068952 | ccRCC1 |
|  | novelGene_5531 | TCONS_00068998 | ccRCC1 |
|  | novelGene_ERV316A3_20q13.33b_AS | TCONS_00072269 | ccRCC3 |
|  | novelGene_MER41_21q22.3b_AS* | TCONS_00073627 | ccRCC1 |
|  | novelGene_MER101_4q23_AS | TCONS_00087928 | ccRCC1 |
|  | novelGene_HERVL32_4q28.1b_AS | TCONS_00087929 | ccRCC1, ccRCC2 |
|  | novelGene_HERVH_7q31.2_AS | TCONS_00106513 | ccRCC2 |
|  | novelGene_ERV316A3_8q21.13c_AS | TCONS_00110959 | ccRCC1, ccRCC3 |
|  | novelGene_PABLA_8p21.3a_AS | TCONS_00110966 | ccRCC1, ccRCC3 |
|  | novelGene_MER4_Xp11.21a_AS | TCONS_00119330 | ccRCC3 |
| **Intersection (n = 1)** | novelGene_ERVLE_7q36.1f_AS | TCONS_00106516 | ccRCC2 |
| **Contains reference (k = 10)** | novelGene_ERVLE_1p31.1j_AS | TCONS_00012666 | ccRCC2 |
|  | novelGene_HML2_10q24.2_AS | TCONS_00018865 | ccRCC3 |
|  | novelGene_HERVL_12p13.1c_AS | TCONS_00031783 | ccRCC3 |
|  | novelGene_MER34B_12q14.3_AS | TCONS_00031764 | ccRCC2 |
|  | novelGene_HERVW_2q31.2_AS | TCONS_00069046 | ccRCC3 |
|  | novelGene_HARLEQUIN_4q13.2b_AS | TCONS_00087954 | ccRCC2 |
|  | novelGene_ERVLB4_4q32.3c_AS | TCONS_00087956 | ccRCC2 |
|  | novelGene_ERV316A3_5p13.3e_AS | TCONS_00094459 | ccRCC1, ccRCC3 |
|  | novelGene_ERVL_6q16.3a_AS | TCONS_00099713 | ccRCC1 |
|  | novelGene_ERVLB4_8p11.22a_AS | TCONS_00110982 | ccRCC2 |

* Two versions of this transcript existed.

**Supplementary Table 1:** HERV isoforms that did not match across four sets of transcriptomes (ccRCC1, ccRCC2, ccRCC3, and Combined).
